# Combined endoscopic drainage for afferent loop obstruction and bilioenteric anastomosis stricture in a patient after pancreatoduodenectomy

**DOI:** 10.1055/a-2346-4685

**Published:** 2024-08-07

**Authors:** Tingting Yu, Suning Hou, Hongfei He, Yaoting Li, Lichao Zhang, Jiao Tian, Senlin Hou

**Affiliations:** 1 71213 Biliopancreatic Endoscopic Surgery Department, The Second Hospital of Hebei Medical University, Shijiazhuang, China

Tumor recurrence is a common cause of bilioenteric anastomosis stricture or afferent loop obstruction (ALO) in patients who have undergone pancreaticoduodenectomy. Endoscopic management in ALO included enteroscopy-assisted luminal stenting with a self-expanding metal stent or plastic stents and endoscopic ultrasound (EUS)-guided enteroenterostomy with a lumen-apposing metal stent 1 2 . In addition, the bridge technique for drainage of the right liver across the left liver through hepaticogastrostomy (HGS) is feasible 3 4 . We describe a case of concurrent ALO and bilioenteric anastomosis stricture in a patient who received the above two endoscopic treatments.

A 55-year-old man was admitted to our hospital with abdominal pain and progressive jaundice. He had undergone a pancreaticoduodenectomy 10 years prior because of distal cholangiocarcinoma. Abdominal computed tomography showed dilated intrahepatic bile ducts and locally dilated intestinal ducts near the bilioenteric anastomosis ( Fig. 1 **a–b** ). We diagnosed ALO, which indirectly caused obstructive jaundice. The patient underwent a gastroscopy; however, due to severe intestinal twisting, the gastroscopy could not reach the site of the afferent loop obstruction. Under fluoroscopy, we attempted to apply a sphincterotome with a guidewire into the obstructed bowel successfully ( Fig. 1 **c–d** ). A 7-Fr plastic stent 73 cm in length (modified nasobiliary tube) was placed to ensure passage through the sharp bends and stricture of the afferent loop ( Fig. 1 **e–f** , Video 1 ). The patient's abdominal pain resolved but jaundice continued to worsen (direct bilirubin up to 357.4 umol/L) after the operation. Subsequently the patient received endoscopic ultrasound-guided hepaticogastrostomy (EUS-HGS) for biliary drainage. We found that the bilioenteric anastomosis was narrow and the guidewire could not pass through. We then bridged a 7-Fr double-pigtail plastic stent 15 cm in length (Zimmon; Wilson-Cook Medical Inc., Limerick, Ireland) to the right intrahepatic bile duct to achieve simultaneous drainage of the right and left intrahepatic bile duct ( Fig. 2 **,** Video 1 ). The patient was discharged as his bilirubin decreased to 284.7 umol/L 10 days later.

**Fig. 1:**
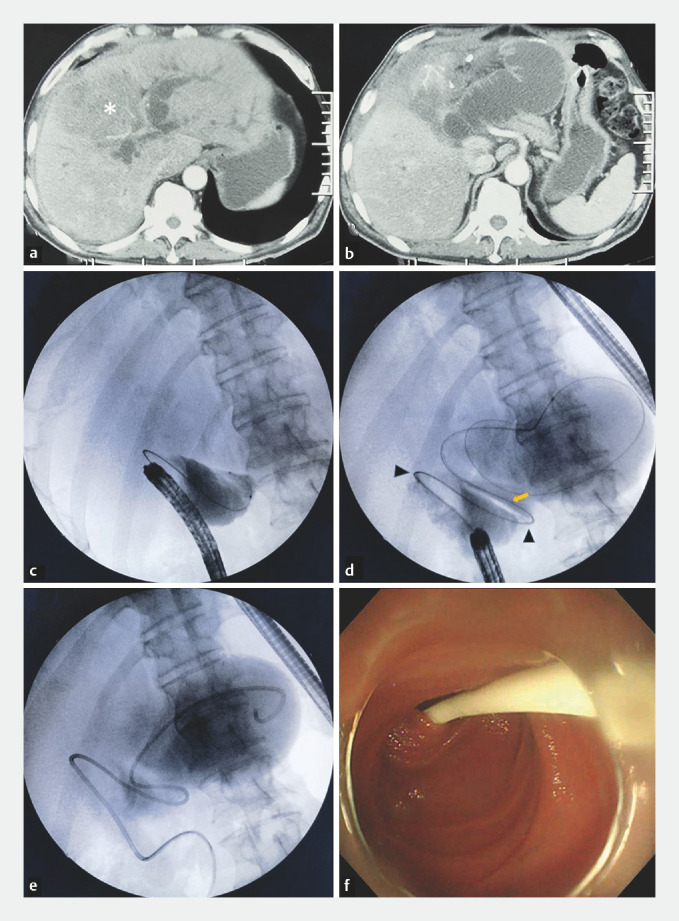
**a** Abdominal computed tomography (CT) showing liver neoplasms (asterisks) and dilated intrahepatic bile ducts. **b** CT showing afferent loop dilatation near the bilioenteric anastomosis. **c** Fluoroscopic image showing the guidewire attempting to enter the obstructed afferent loop with the assistance of the sphincterotome. **d** The guidewire passed through sharp bends (triangles) and the stricture (arrow) of the afferent loop. **e** A self-made 7-Fr plastic stent 70 cm in length was inserted into the dilated afferent loop. **f** Endoscopy showing the properly placed plastic stent.

**Fig. 2:**
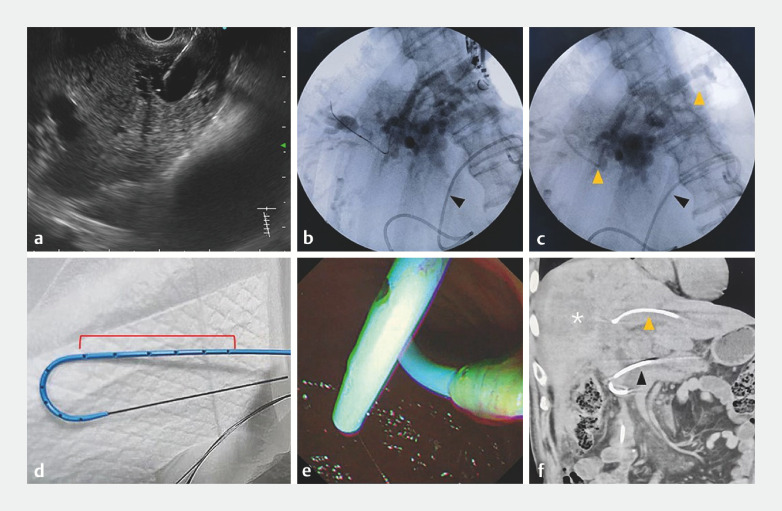
**a** Endoscopic ultrasound (EUS) image shows the left intrahepatic bile duct (segment 2) was punctured using a 19G fine-needle aspiration needle. **b** Fluoroscopic image showing a guidewire entered from the left intrahepatic bile duct to the right intrahepatic bile duct, and a plastic stent was placed in the dilated afferent loop (black triangle). **c** A 7-Fr double-pigtail plastic stent 15 cm in length was inserted into the left and right hepatic ducts (yellow triangles). **d** Side holes were added on the plastic stent. **e** Endoscopy shows the plastic stent placed to drain bile. **f** CT showing improved intrahepatic bile duct obstruction and afferent loop dilatation.

Combined gastroscopy-assisted luminal stenting and endoscopic ultrasound-guided hepaticogastrostomy with bridge technique for afferent loop obstruction and bilioenteric anastomosis stricture in a patient after pancreatoduodenectomy.Video 1

Endoscopy_UCTN_Code_TTT_1AS_2AH

## Notes

Endoscopy E-Videos https://eref.thieme.de/e-videos
*E-Videos* is an open access online section of the journal *Endoscopy* , reporting on interesting cases and new techniques in gastroenterological endoscopy. All papers include a high-quality video and are published with a Creative Commons CC-BY license. Endoscopy E-Videos qualify for HINARI discounts and waivers and eligibility is automatically checked during the submission process. We grant 100% waivers to articles whose corresponding authors are based in Group A countries and 50% waivers to those who are based in Group B countries as classified by Research4Life (see: https://www.research4life.org/access/eligibility/ ). This section has its own submission website at https://mc.manuscriptcentral.com/e-videos .
